# Changes of Blood Flow Volume in the Superior Mesenteric Artery and Brachial Artery with Abdominal Thermal Stimulation

**DOI:** 10.1093/ecam/nep110

**Published:** 2011-02-13

**Authors:** Shin Takayama, Takashi Seki, Masashi Watanabe, Shigeru Takashima, Norihiro Sugita, Satoshi Konno, Takashi Takeda, Hiroyuki Arai, Tomoyuki Yambe, Nobuo Yaegashi, Makoto Yoshizawa, Shigenao Maruyama, Shin-Ichi Nitta

**Affiliations:** ^1^Center for Asian Traditional Medicine, Graduate School of Medicine, Tohoku University, Miyagi, Japan; ^2^Institute of Fluid Science, Tohoku University, Miyagi, Japan; ^3^Department of Electrical and Communication Engineering, Graduate School of Engineering, Tohoku University, Miyagi, Japan; ^4^Institute of Development, Aging and Cancer, Tohoku University, Miyagi, Japan; ^5^Research Division on Advanced Information Technology, Cyberscience Center, Tohoku University, Miyagi, Japan

## Abstract

In traditional Chinese medicine, moxibustion is a local thermal therapy that is used for several conditions. Quantifying the effects of moxibustion therapy has been difficult because the treatment temperature depends on the physician's experience, and the temperature distribution in the target area is not uniform. This prospective observational study aims to quantify the effect of local thermal stimulation to the abdomen. We developed a heat transfer control device (HTCD) for local thermal stimulation. Twenty-four healthy subjects were enrolled and they underwent abdominal thermal stimulation to the para-umbilical region with the device for 20 min. Blood flow volume in the superior mesenteric artery (SMA) and brachial artery (BA), the heart rate and the blood pressure were measured at rest, 15 min after starting thermal stimulation and 10, 20, 30 and 40 min after completing thermal stimulation. Blood flow parameters were measured by high-resolution ultrasound. In the SMA, blood flow volume was significantly increased during thermal stimulation (*P* < .01), as well as at 10 min (*P* < .01) and 20 min (*P* < .05) after stimulation. In the BA, blood flow volume decreased at 40 min after stimulation (*P* < .01). In conclusion we could quantify the effect of local thermal stimulation with an HTCD and high-resolution ultrasound. Thermal stimulation of the para-umbilical region increased blood flow in the SMA 20 min after stimulation in healthy subjects.

## 1. Introduction

Recently, thermal therapy has been used for a number of conditions such as inflammatory bowel diseases, chronic heart failure, chronic pain, depressive state and chronic obstructive pulmonary disease [1–13]. It can be classified into two types: one type involves heating the whole body or a large part of it in a sauna or warm bath. This procedure has been shown to reduce cardiac after-load and pre-load and to increase cardiac function in patients with chronic heart failure [4–6, 12]. The other type involves heating of a local body area. Local thermal therapy with a hot pack or paraffin has often been used to treat pain [8]. It has been reported that local thermal therapy reduces myotonia, improves the circulation and relieves pain by accelerating the removal of pain-producing substances. In traditional Chinese medicine, a local thermal therapy known as moxibustion is widely used for several conditions such as pain, nausea, vomiting, neurodegenerative diseases, inflammatory bowel diseases and cerebrovascular and cardiovascular diseases [14–16]. Moxibustion is the way of heating a local area in which moxa is burned at acupoints on the skin. Because the direct burning of moxa on the skin can be dangerous, materials like salt, ginger or garlic may be used as a buffer between the skin and the moxa. Several previous studies have assessed the effect of moxibustion [1–3, 17], but controlling the temperature achieved by moxibustion has been difficult. The actual temperature depends on the physician's experience and the temperature distribution in the target area is not uniform. Therefore, we developed the heat transfer control device (HTCD) used in the present study.

Under normal conditions, the superior mesenteric artery (SMA) blood flow pattern and velocity show large variations due to the metabolic activity of the bowel [18–20]. The SMA blood flow volume also changes in several diseases [21–23]. The blood flow volume is also strongly related to mesenteric ischemia, especially chronic mesenteric ischemia [24].

There have been no reports about SMA blood flow changes in relation to moxibustion therapy. Therefore, this study was performed to evaluate the changes of SMA blood flow volume with abdominal ultrasound after thermal stimulation with an HTCD.

## 2. Methods

### 2.1. Subjects

We enrolled 24 healthy male subjects, who had a mean ± SD age of 31.2 ± 6.9 years (range 21–44 years). The study protocol was approved by the Ethics Committee of Tohoku University Graduate School of Medicine. Written informed consent to participation was given by all subjects.

### 2.2. Thermal Stimulation Method

**Figure 1:**
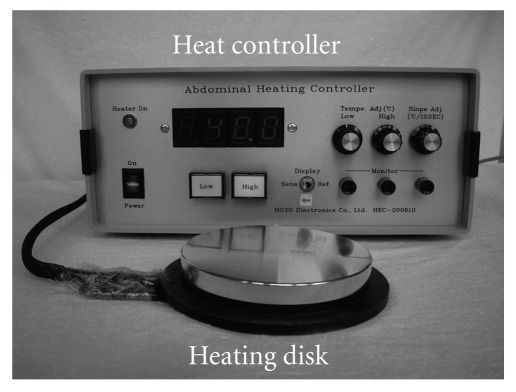
HTCD is composed of a heating disk, temperature sensor and a heat controller.

To quantify the heat delivered, we developed an HTCD instead of moxibustion for local thermal stimulation (Figure 1) [25]. This device was composed of a heating disk (10 cm in diameter), a temperature sensor and a heat controller. The temperature of the heating disk could be increased incrementally to the target and would not exceed it. We could control the temperature of the heating disk with a precision of 0.1°C and the device heated a small region to a uniform temperature. Therefore, it was suitable to quantify the effect of thermal stimulation. Thermal damage occurs when the tissue temperature rises above 44°C [26, 27], so the temperature of the disk was set at 40–41°C for safety. A patent for this device is pending in Japan.

### 2.3. Study Protocol

**Figure 2:**
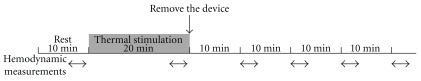
Outline of the study. Thermal stimulation was applied to the para-umbilical region with an HTCD for 20 min. Hemodynamics were measured at rest (baseline), during thermal stimulation (after 15 min) and at 10, 20, 30 and 40 min after the completion of thermal stimulation.

This is a prospective observational study. We performed abdominal thermal stimulation at the para-umbilical region with an HTCD for 20 min and measured SMA hemodynamics by ultrasound from rest until 40 min after thermal stimulation. To compare intestinal and peripheral blood flow volume, we measured the hemodynamics of the brachial artery (BA) simultaneously. An outline of the study is shown in Figure 2.

**Figure 3:**
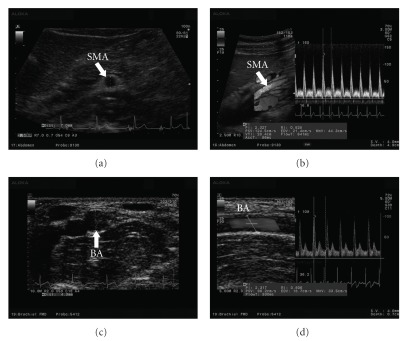
Hemodynamic data obtained by ultrasound. (a) VD of the SMA, (b) blood flow velocity of the SMA, (c) vessel diameter of the BA and (d) blood flow velocity of the BA.

All subjects were examined in the morning after an overnight fast (at least 8 h). The subject rested in the supine position in a quiet, air-conditioned room (temperature 25-26°C). Three monitoring electrocardiographic electrodes were attached to the chest. Blood pressure was measured in the left upper arm with an oscillometer (HEM-9000AI, Omron Healthcare Co., Ltd, Kyoto, Japan). SMA and right BA hemodynamics were measured with an ultrasound system (Prosound *α*10, Aloka Co., Ltd, Tokyo, Japan). High-resolution ultrasound combined with pulsed Doppler is useful to investigate small vessels, such as the coronary, splenic, adrenal and SMA [28]. The present ultrasound system had a 5 MHz convex transducer and a 13 MHz linear transducer. We used the convex transducer to measure SMA hemodynamics and the linear transducer for BA hemodynamics. The cross-sectional areas (CSAs) of the SMA and BA were calculated by vessel diameter (VD), the distance from anterior to posterior intima (Figures 3(a) and 3(c)). As described earlier, SMA measurements were acquired within 2-3 cm of the origin of the artery (Figure 3(b)) [29, 30]. Pulsed Doppler signals were obtained at the same site. The BA was monitored at a site above the elbow (Figure 3(d)). For accurate measurement, a Doppler angle of 60° or less was employed [31, 32]. Each Doppler waveform was drawn automatically and calculated by using the software in the ultrasound system. The following hemodynamic parameters were determined.

Hemodynamic parameters:

- VD
- CSA = (VD/2)^2^ × **π**
- Peak systolic velocity (PSV)
- End-diastolic velocity (EDV)
- Resistive index (RI) = (PSV−EDV)/PSV
- Pulsatility index (PI) = (PSV−EDV)/MV
- Mean flow velocity (MV)
- Blood flow volume = CSA × MV [29, 30]

Each parameter was recorded three times in three different cardiac cycles and averaged for each subject in an effort to minimize random errors [29].

After positioning the ultrasound system, the subjects rested in the supine position for 10 min. Abdominal thermal stimulation was done at the para-umbilical region with the HTCD for 20 min from a temperature of 40°C. After 5 min, if subjects were used to the heat, the temperature was increased to 41°C. After thermal stimulation for 20 min, the device was removed.

We measured the SMA and BA hemodynamics, heart rate and blood pressure at rest (baseline), after 15 min of thermal stimulation, and 10, 20, 30 and 40 min after the end of thermal stimulation (Figure 2).

### 2.4. Statistical Analysis

Statistical analysis was performed with SPSS software (version 16.0, SPSS Japan Inc., Tokyo, Japan). Repeated measures analysis of variance with a Tukey post hoc test was used for statistical comparison with baseline. Although 24 subjects were enrolled in our study, three subjects were excluded because their examinations were technically unsuccessful (the SMA could not be identified clearly due to intestinal gas). As a result, 21 subjects were included in the final analysis. Results are presented as the medians and quartile (first and third), the means and SEM and 95% confidence intervals. *P* < .05 was used to indicate significance in all statistical tests.

## 3. Results

### 3.1. Hemodynamic Parameters in the SMA and BA

The hemodynamic parameters in the SMA and BA are summarized in Tables 1 and 2 .

**Figure 4:**
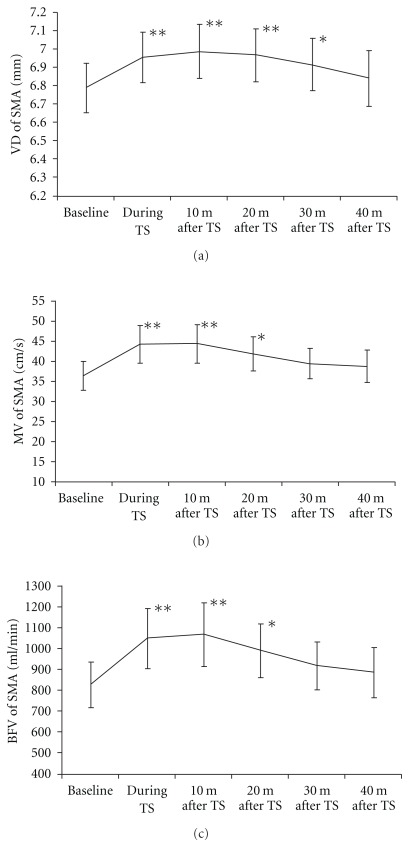
Hemodynamic changes in the SMA. (a) Change of vessel diameter, (b) change of MV and (c) change of blood flow volume. The values represent the means and SEM. *P < .05,**P < .01 versus baseline.

**Figure 5:**
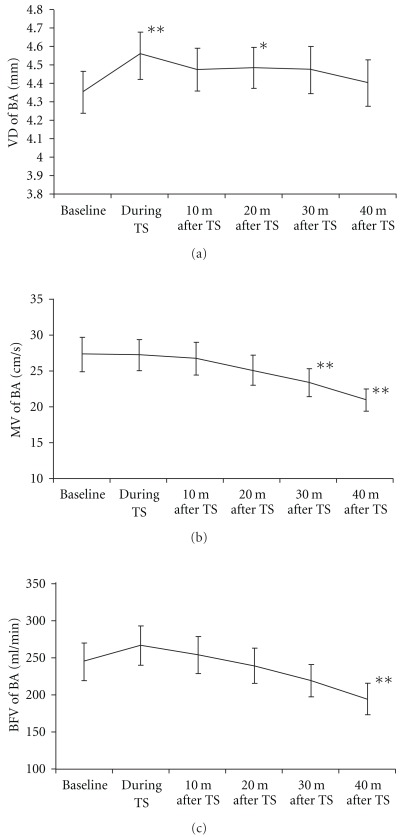
Hemodynamic changes in the BA. (a) Change of vessel diameter, (b) change of mean flow velocity and (c) change of blood flow volume. The values represent the means and SEM. *P < .05, **P < .01 versus baseline.

The changes of VD, MV and blood flow volume in the SMA and BA are shown in Figures 4(a) and 5(a), 4(b) and 5(b) and 4(c) and 5(c), respectively. The SMA diameter significantly increased during thermal stimulation (*P* < .01), as well as at 10 min (*P* < .01), 20 min (*P* < .01) and 30 min (*P* < .05) after thermal stimulation (Figure 4(a)). The BA diameter also increased significantly during thermal stimulation (*P* < .01) and at 20 min (*P* < .05) after thermal stimulation (Figure 5(a)). The MV in the SMA showed significant increase during thermal stimulation (*P* < .01), as well as at 10 min (*P* < .01) and 20 min (*P* < .01) after the end of thermal stimulation (Figure 4(b)), but the flow velocity in the BA significantly decreased at 30 min (*P* < .01) and 40 min (*P* < .01) after thermal stimulation (Figure 5(b)). The blood flow volume in the SMA significantly increased during thermal stimulation (*P* < .01), as well as at 10 min (*P* < .01) and 20 min (*P* < .05) after the end of thermal stimulation (Figure 4(c)). In the BA, however, blood flow volume decreased at 40 min after the end of thermal stimulation (*P* < .01) (Figure 5(c)).

PSV in the SMA showed a significant increase during thermal stimulation (*P* < .01) (Table 1), but while it significantly decreased in the BA at 30 min (*P* < .05) and 40 min (*P* < .01) after the end of thermal stimulation (Table 2). EDV did not change in the SMA (Table 1), but it decreased significantly at 30 min (*P* < .01) and 40 min (*P* < .01) after the end of thermal stimulation in the BA (Table 2). The RI in the SMA did not change (Table 1), but it significantly increased in the BA at 40 min after the end of thermal stimulation (*P* < .01) (Table 2). However, the PI showed no significant changes in the SMA and BA compared with baseline (Tables 1 and 2).

### 3.2. Systemic Hemodynamics

The blood pressure and heart rate are shown in Table 3. There were no significant changes of heart rate compared with baseline. Systolic blood pressure showed a significant increase at 40 min after the end of thermal stimulation (*P* < .05), but there no significant changes in diastolic blood pressure was observed between baseline and 40 min after thermal stimulation.

### 3.3. Side Effects of Abdominal Thermal Stimulation

There were no complications such as local burns, pain, discomfort or other problems that needed treatment.

## 4. Discussion

This is the first report about the changes of blood flow volume for SMA and BA by local thermal stimulation with an ultrasound system. In SMA, blood flow volume significantly increased during thermal stimulation, as well as at 10 and 20 min after stimulation. In BA, blood flow volume significantly decreased 40 min after stimulation.

**Figure 6:**
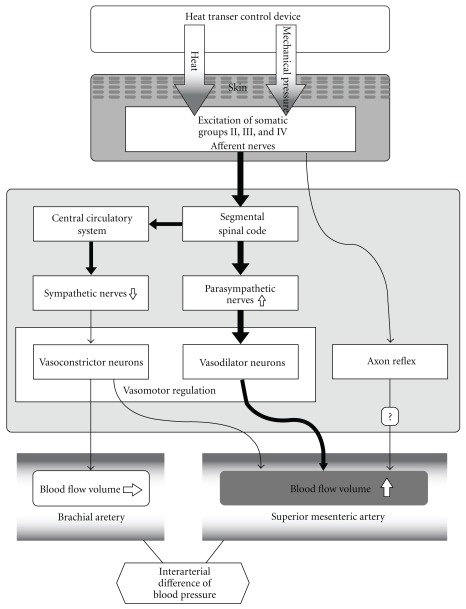
Hypothesis of mechanisms for increasing blood flow volume in SMA compared with BA.

We expressed the changes of SMA hemodynamics with an ultrasound system. The SMA supplies blood to the duodenum, small bowel, colon and rectum. Blood flow volume in the SMA can be altered by several diseases, such as Crohn's disease, ulcerative colitis, enteroperitoneal tuberculosis and appendicitis [21–23, 28]. Blood flow volume is also altered in mesenteric ischemia, with chronic mesenteric ischemia being closely related to SMA blood flow [24]. After meals, there is an increase of mesenteric blood flow to assist digestion [20] and vasodilation allows more blood flow into the intestines. The pulsed Doppler method has been used to assess rapid changes of hemodynamics in the SMA. The blood flow volume increases significantly after food intake, whereas exercise reduces mesenteric blood flow in normal subjects [20, 33]. Various vasoconstrictive stimuli, including mental arithmetic, cold, isometric exercise and head-up tilt also influence the SMA diameter and blood flow volume [34]. However, it has not been previously shown that SMA blood flow volume is increased by local thermal stimulation. Uchida et al. [35] investigated the mechanisms by which blood flow was increased in the internal organs by acupuncture. They suggested that the increase of uterine blood flow with acupuncture was mediated by a segmental spinal reflex mechanism that involved excitation of somatic group II, III and IV afferents and pelvic parasympathetic cholinergic nerves. It has been reported that several acupoints have an influence on the gastrointestinal motility [36], which is thought to be mediated via parasympathetic nerves [37, 38]. Thermal stimulation of the para-umbilical region simultaneously stimulates multiple acupoints related to the intestine by thermal energy and mechanical pressure. There are several acupoints (including CV-7, CV-8, CV-9 and ST-25) located at the para-umbilical region where we performed abdominal thermal stimulation. In particular, CV-8 and ST-25 are considered to influence the stomach, spleen and intestine in traditional Chinese medicine [39]. Thermal stimulation and pressure applied to the skin by the present method influences somatic afferent fibers. Thus, we speculate that the present method of stimulation increase SMA blood flow mediated by somatic group II, III and IV afferent fibers and parasympathetic cholinergic nerves. Our hypothesis for the mechanism of increase of blood flow volume in SMA by HTCD is shown in Figure 6.

The SMA blood flow reflects mesenteric flow, whereas BA blood flow reflects peripheral flow. The present study showed that the blood flow volume of the SMA increased significantly during thermal stimulation and up to 20 min after thermal stimulation, while it decreased 40 min after thermal stimulation in the BA. According to the blood flow velocity curve of the SMA and BA, the interarterial differences of blood pressure between SMA and BA can be calculated. The difference significantly increased during thermal stimulation (95% confidence interval (95% CI) 0.5–3.9 mmHg; *P* < .01), at 10 min (95% CI 0.3–3.6; *P* < .01) and 20 min (95% CI 0.17–3.6; *P* < .05) after thermal stimulation compared with baseline. Thus, we can speculate that the interarterial difference of blood pressure is one of the causes of the shift in blood volume. Tsuru et al. suggested that acupuncture causes vasodilation and increases blood flow in various organs by modulating the central circulatory system and axon reflexes, with the effect depending on the site of stimulation [40]. It seems that the SMA (near the stimulated area) was influenced by a vasodilatory action, while the BA (distant from the stimulated area) was little influenced by that mechanism (Figure 6). Vasomotor regulation of blood VD induces changes in blood flow. In the extremities, vasoconstriction is more important than active vasodilatation to regulate the systemic circulation [41]. Vasomotor function in the hand is mainly controlled by sympathetic nerves [42, 43], and it has been reported that the distribution of vasodilator neurons is not uniform and that the neurons innervating vascular beds perform different functions [44]. Thus, there are the difference of the distribution of vasoconstrictor and vasodilator neurons between SMA and BA. The difference in blood flow volume changes between the SMA and BA may be also related to the different distribution of these neurons (Figure 6).

Our hypothesis is that the change of blood flow volume by thermal stimulation with HTCD is related not to the direct thermal energy, but to the reaction of vasodilator neurons. The thermal energy in the skin is continuously carried off by blood circulation in the skin, fat and muscles in the human body. An increase in the tissue temperature causes an activation of the metabolism. The increase in the metabolism and subsequent increase in perfusion due to increasing temperatures are modeled according to a well-known *Q*_10_ relation of thermal physiology [45]. This relation states that for every 10°C increase in tissue temperature, there is a corresponding increase in cell metabolism and blood flow with a constant factor *Q*_10_ [46]:

(1) $$\begin{array}{c} \omega_{b}(T)=\omega_{0}Q_{10}^{(T-T_{a})/10}\\ {\overset{˙}{q}}_{met}(T)={\overset{˙}{q}}_{0}Q_{10}^{(T-T_{a})/10} \end{array}$$

where *T* (°C) is the temperature, *T*_a_ is the temperature at artery, **ω**_b_ (s^−1^) is the blood flow rate of a tissue, *q*_met_ is metabolic heat generation. **ω**_0_ is the perfusion rate under normal condition at *T*_0_, and *q*_0_ is the metabolic heat generation under normal condition. To clarify whether thermal stimulation can heat the internal organ directly, we previously analyzed the distribution of temperature in the subcutaneous tissue [47]. In most bioheat transfer analysis, a continuous model proposed by Pennes is widely used. The model is given by [48]:

(2) $$\begin{array}{cc} \rho c\frac{\partial T}{\partial t} & =\frac{1}{r}\frac{\partial }{\partial r}(kr\frac{\partial T}{\partial r})+\frac{\partial }{\partial z}(k\frac{\partial T}{\partial z})\\ & \;+\rho_{b}c_{b}\omega_{b}(T)(T_{a}-T)+{\overset{˙}{q}}_{\text{met}}(T), \end{array}$$

where **ρ** (kg m^−3^) is density, *c* (J kg^−1^ °C^−1^) is specific heat, *k* (W m^−1^ °C^−1^) is thermal conductivity, *t* (s) is time and subscript b expresses blood. The expression on the left means a change in temperature. The first to third expressions on the right show heat conduction and heat exchange between blood and tissue, while the fourth shows metabolic heat generation. We developed a model to analyze the distribution of temperature achieved with local thermal stimulation and this model was composed of a skin layer (0.4 mm), a fat layer (7.8 mm) and a muscle layer (13.4 mm) [49]. Our analysis indicated that heat was almost dissipated in the lipid layer and little heat penetrates to the internal organs. Thus, if we heat the surface of the abdomen to 42°C for 15 min, the intestine is not heated directly. Therefore, the blood flow volume increase observed with the local thermal stimulation is not simply due to the transfer of thermal energy, but due to the fact that heat stimulation leads to hemodynamic changes.

In the present study, we demonstrated the change of blood flow volume by HTCD. To our knowledge, this is the first report about the effect of abdominal thermal stimulation on blood flow in the SMA and BA. This trial was a pilot study with a small sample size, and no control and randomization. The subjects were all men. However, the pattern of change of blood flow volume in SMA and BA was same in the subjects, except for one. We supposed that the blood flow volume did not occur by chance. We would like to undertake this study further in both men and women, with a larger sample size, control group and randomization.

Mesenteric ischemia results from decreased blood flow to the bowel, causing several symptoms such as pain, nausea and vomiting. Non-occlusive mesenteric ischemia is an acute mesenteric circulatory disorder which is induced by vasospasm [50]. Chronic mesenteric ischemia usually is caused by atherosclerosis [51]. In these conditions, the pathophysiology is same as the mesenteric ischemia. The treatment of mesenteric ischemia is the reperfusion by drugs or vessel reconstruction. Sometimes vasodilative drugs are selected for conservative treatment [50–52]. We demonstrated the increase of SMA blood flow volume with local thermal therapy in the present study. The treatment of local thermal therapy with HTCD may be useful for mesenteric ischemia to increase the blood flow volume.

When SMA hemodynamics during thermal stimulation was measured by ultrasound, the acceleration of intestinal peristalsis was observed along with an increase in blood-flow volume. Thus, thermal stimulation not only increases the blood-flow volume, but also improves intestinal motility. Abdominal thermal therapy may be useful for patients with low SMA blood flow, paralytic ileus or chronic constipation. In the future, we hope to study the effect of thermal stimulation on patients with such disorders.

Moxibustion is often combined with acupuncture; however, in the West it is not often used because of the odor from the burning moxa. HTCD can heat the target area uniformly without any odor or the dangers associated with a fire. Thus, abdominal thermal therapy with an HTCD may be useful for patients with intestinal disorders instead of moxibustion. We would like to undertake further studies to clarify the effect of local thermal stimulation, for not only the abdomen but also for other areas.

In conclusion, we could measure the effect of local thermal stimulation quantitatively with an HTCD and high-resolution ultrasound. Thermal stimulation of the para-umbilical region could increase blood flow in the SMA 20 min after stimulation in healthy subjects.

## Funding

Special Coordination Funds for Promoting Science and Technology from the Japanese Ministry of Education, Culture, Sports, Science and Technology.

## Figures and Tables

**Table 1:** Summary of hemodynamic parameters.

Parameter	Baseline	During TS	10 min after TS	20 min after TS	30 min after TS	40 min after TS
VD (mm)						
Median (first, third quartile)	6.87 (6.4, 7.17)	7.0 (6.67, 7.3)	7.05 (6.65, 7.53)	7.13 (6.7, 7.47)	7.0 (6.6, 7.2)	6.97 (6.55, 7.3)
Mean (SEM)	6.79 (0.14)	6.69 (0.14)**	7.01 (0.15)**	6.97 (0.15)**	6.92 (0.14)*	6.84 (0.15)
95% CI	6.52–7.06	6.69–7.23	6.70–7.28	6.68–7.26	6.64–7.20	6.55–7.14
PSV (cm/s)						
Median (first, third quartile)	144.3 (121.3, 168.2)	163.3 (146.3, 194.2)	166.7 (149.6, 191.7)	153.4 (139.6, 205.7)	156.75 (132.5, 181.8)	156.4 (134.8, 192.6)
Mean (SEM)	151.3 (9.1)	178.3 (1.04)**	170.0 (12.1)	169.8 (9.8)	162.4 (9.7)	161.2 (8.5)
95% CI	133.5–169.1	157.9–198.8	146.4–193.7	150.6–189.1	143.4–181.4	145.0–177.6
EDV (cm/s)						
Median (first, third quartile)	6.5 (1.35, 13.9)	10.7 (5.9, 15.8)	8.7 (2.2, 13.2)	9.9 (0.3, 14.7)	6.6 (0.0, 14.8)	7.4 (0.0, 13.6)
Mean (SEM)	10.7 (2.7)	14.9 (3.7)	12.3 (3.8)	12.0 (3.2)	10.5 (2.8)	11.0 (3.1)
95% CI	5.3–16.2	7.6–22.1	4.9–19.7	5.8–18.3	5.0–16.0	5.1–17.0
RI						
Median (first, third quartile)	1 (0.94, 1.04)	0.98 (0.92, 1.00)	0.96 (0.92, 1.00)	0.97 (0.93, 1.00)	1 (0.92, 1.00)	0.98 (0.92, 1.00)
Mean (SEM)	0.98 (0.02)	0.96 (0.02)	0.95 (0.01)	0.96 (0.02)	0.97 (0.02)	0.96 (0.02)
95% CI	0.95–1.01	0.92–1.00	0.92–0.98	0.93–0.99	0.94–1.00	0.93–0.99
PI						
Median (first, third quartile)	4.57 (3.00, 5.26)	4 (3.41, 4.93)	3.98 (3.23, 4.83)	3.84 (3.43, 4.99)	4.16 (3.48, 4.95)	4 (3.39, 5.35)
Mean (SEM)	4.29 (0.40)	4.21 (0.28)	4.11 (0.26)	4.30 (0.29)	4.30 (0.27)	4.36 (0.32)
95% CI	3.51–5.07	3.66–4.76	3.60–4.61	3.73–4.87	3.77–4.84	3.74–4.98
MV (ml/s)						
Median (first, third quartile)	34.4 (28.5, 38.6)	39.8 (34.2, 44.3)	38.6 (31.7, 45.7)	36.7 (30.1, 46.4)	33.5 (29.5, 41.3)	33.5 (28.9, 38.7)
Mean (SEM)	36.6 (3.6)	44.5 (4.6)**	44.6 (4.8)**	42.0 (4.3)*	49.66 (3.7)	39.0 (3.9)
95% CI	29.6–43.7	35.5–53.5	35.1–54.1	33.6–50.3	32.3–47.0	31.5–46.6
BFV (ml/min)						
Median (first, third quartile)	747.1 (565.1, 891.6)	814 (755.0, 1066.7)	847.3 (684.6, 1098.8)	827.7 (633.7, 1058.7)	818 (566.0, 960.3)	751.6 (577.5, 859.3)
Mean (SEM)	822.1 (109.3)	1045.0 (143.5)**	1062.8 (153.6)**	986.3 (129.8)*	913.7 (11.7)	882.1 (119.3)
95% CI	623.7–1020.4	784.6–1035.4	784.1–1341.6	750.8–1221.8	711.0–116.3	671.7–1092.5

Hemodynamic parameters measured by ultrasound are shown for the SMA. The values represent medians and quartile (first and third), means and SEM and 95% CI. TS, thermal stimulation; BFV, blood flow volume. *P < .05, **P < .01 versus baseline.

**Table 2:** Summary of hemodynamic parameters.

Parameter	Baseline	During TS	10 min after TS	20 min after TS	30 min after TS	40 min after TS
VD (mm)						
Median (first, third quartile)	4.4 (3.85, 4.6)	4.6 (4.0, 4.8)	4.47 (4.07, 4.77)	4.45 (4.0, 4.73)	4.55 (3.87, 4.7)	4.45 (3.85, 4.76)
Mean (SEM)	4.35 (0.11)	4.56 (0.13)**	4.47 (0.12)	4.48 (0.11)*	4.47 (0.13)	4.40 (0.13)
95% CI	4.13–4.58	4.31–4.80	4.24–4.71	4.23–4.70	4.22–4.72	4.16–4.64
PSV (cm/s)						
Median (first, third quartile)	76.8 (64.9, 95)	77.8 (70, 87.8)	82.1 (62, 93)	74.375 (58.2, 82.2)	74.7 (62.2, 87.6)	73 (57.5, 78.1)
Mean (SEM)	80.2 (4.0)	80.0 (4.0)	79.0 (4.3)	74.4 (3.5)	73.3 (3.5)*	70.1 (3.5)**
95% CI	7.32–88.0	72.1–87.9	70.6–87.4	67.5–81.3	66.5–80.2	63.3–76.9
EDV (cm/s)						
Median (first, third quartile)	16.7 (14.0, 21.2)	16.9 (14.7, 20.8)	16.6 (12.2, 20.7)	14.8 (12.0, 20.2)	14.2 (12.2, 17.1)	11.3 (9.3, 14.2)
Mean (SEM)	17.9 (1.3)	17.3 (1.2)	16.8 (1.2)	15.6 (1.1)	14.8 (1.1)**	11.6 (0.9)**
95% CI	15.5–20.4	15.0–19.6	14.4–19.2	13.4–17.8	12.6–17.1	9.9–13.3
RI						
Median (first, third quartile)	0.83 (0.75, 0.90)	0.80 (0.75, 0.86)	0.79 (0.76, 0.85)	0.81 (0.78, 0.85)	0.83 (0.80, 0.96)	0.89 (0.95)
Mean (SEM)	0.87 (0.04)	0.85 (0.03)	0.85 (0.04)	0.87 (0.04)	0.90 (0.04)	0.92 (0.03)**
95% CI	0.79,–0.94	0.77–0.92	0.78–0.92	0.79–0.94	0.82–0.98	0.87–0.98
PI						
Median (first, third quartile)	2.45 (1.98, 3.18)	2.33 (1.97, 2.68)	2.25 (1.96, 2.85)	2.33 (2.05, 2.88)	2.64 (2.20, 3.48)	3.26 (2.77, 3.76)
Mean (SEM)	4.22 (1.05)	3.29 (0.71)	3.32 (0.67)	3.58 (0.83)	3.65 (0.69)	3.62 (0.50)
95% CI	2.17–6.27	1.90–4.68	1.99–4.63	1.96–5.21	2.29–5.01	2.66–4.58
MV (ml/s)						
Median (first, third quartile)	28.4 (20.7, 34.3)	29.0 (19.4, 34.6)	28.8 (20.4, 33.0)	25.9 (19.9, 32.2)	24.5 (16.2, 28.7)	22.0 (15.5, 25.4)
Mean (SEM)	27.4 (2.4)	27.3 (2.2)	26.8 (2.3)	25.1 (2.1)	23.4 (1.9)**	20.9 (1.6)**
95% CI	22.6–32.1	23.0–31.5	22.3–31.2	21.1–29.2	19.5–27.2	17.9–23.9
BFV (ml/min)						
Median (first, third quartile)	248.2 (204.3, 326.3)	262.8 (211.1, 365.8)	271.3 (190.8, 329.2)	242.7 (188.4, 314.0)	218.4 (154.0, 273.4)	178.2 (136.6, 247.1)
Mean (SEM)	246.3 (25.2)	268.2 (26.4)	255.4 (25.2)	240.4 (24.1)	220.8 (22.2)	195.3 (21.1)**
95% CI	200.6–292.0	220.3–316.1	209.8–301.1	196.8–284.0	180.5–261.1	158.1–232.5

Hemodynamic parameters measured by ultrasound are shown for the brachial artery. The values represent medians and quartile (first and third), means and SEM and 95% CI. TS, thermal stimulation; BFV, blood flow volume. *P < .05,**P < .01 versus baseline.

**Table 3:** Summary of hemodynamic parameters.

Parameter	Baseline	During TS	10 min after TS	20 min after TS	30 min after TS	40 min after TS
HR (beats/min)						
Median (first, third quartile)	61 (54, 64)	57 (54, 67)	58 (54, 61)	56 (53, 61)	56 (54, 62)	58 (53.3, 60.5)
Mean (SEM)	59.6 (1.9)	60.0 (2.0)	58.5 (1.6)	57.3 (1.8)	58.7 (1.7)	58 (1.8)
95% CI	56.0–63.3	56.1–63.8	55.2–61.7	53.9–60.8	55.4–62.0	54.8–61.54
Syst BP (mmHg)						
Median (first, third quartile)	115 (106, 125)	116 (109, 125)	114 (108, 127)	118 (111, 124)	116 (113, 125)	118.5 (112, 127)
Mean (SEM)	117.3 (2.4)	117.9 (2.6)	117.5 (2.6)	118.9 (2.5)	120.4 (2.9)	121.9 (3.1)*
95% CI	113.0–121.5	113.5–122.2	113.2–121.7	114.3–123.6	115.6–125.5	117.1–126.7
Dia BP (mmHg)						
Median (first, third quartile)	69 (63, 75)	66 (63, 80)	66 (62, 72)	68 (62, 78)	69 (62, 76)	69.5 (63.8, 77.3)
Mean (SEM)	69.8 (2.2)	69.9 (2.2)	68.1 (2.2)	70.6 (2.4)	70.7 (2.6)	72.1 (2.5)
95% CI	65.5–74.0	65.6–74.2	63.9–72.4	66.0–75.2	65.5–75.8	67.3–76.9

Blood pressure and heart rate are also shown. The values represent medians and quartile (first and third), means and SEM and 95% CI. TS, thermal stimulation; HR, heart rate; Syst BP, systolic blood pressure; Dia BP, diastolic blood pressure; CI, confidence interval. *P < .05, **P < .01 versus baseline.
